# Smart imaging to empower brain-wide neuroscience at single-cell levels

**DOI:** 10.1186/s40708-022-00158-4

**Published:** 2022-05-11

**Authors:** Shuxia Guo, Jie Xue, Jian Liu, Xiangqiao Ye, Yichen Guo, Di Liu, Xuan Zhao, Feng Xiong, Xiaofeng Han, Hanchuan Peng

**Affiliations:** Institute for Brain and Intelligence, Southeast University, Nanjing, 210096 Jiangsu China

**Keywords:** Smart imaging, Neuroscience, Artificial intelligence, Brain-wide, Single-cell

## Abstract

A deep understanding of the neuronal connectivity and networks with detailed cell typing across brain regions is necessary to unravel the mechanisms behind the emotional and memorial functions as well as to find the treatment of brain impairment. Brain-wide imaging with single-cell resolution provides unique advantages to access morphological features of a neuron and to investigate the connectivity of neuron networks, which has led to exciting discoveries over the past years based on animal models, such as rodents. Nonetheless, high-throughput systems are in urgent demand to support studies of neural morphologies at larger scale and more detailed level, as well as to enable research on non-human primates (NHP) and human brains. The advances in artificial intelligence (AI) and computational resources bring great opportunity to ‘smart’ imaging systems, i.e., to automate, speed up, optimize and upgrade the imaging systems with AI and computational strategies. In this light, we review the important computational techniques that can support smart systems in brain-wide imaging at single-cell resolution.

## Introduction

To understand the brain structures is one of the primary targets of modern science. Among those the shapes of neurons play a fundamental role. The function of a neuron both dictates and is constrained by its morphology and connection with other neurons. The neuron circuits and connectivity provide scientific evidence and basis to understand the emotional and memorial activities and the brain diseases [1–4]. It is of uttermost importance to accurately locate and identify the neural morphologies at the scale of the entire brain. Under this light, brain initiatives have been announced to build brain-wide atlases to unravel the neuronal connectivity and neural circuits, including the U.S. BRAIN Initiative [5, 6], Europe’s Human Brain Project [7], and China’s Brain Project [8]. These projects are expected to facilitate the treatment of neurological and psychiatric disorders and to promote new breakthroughs in neuromorphic computing and artificial intelligence. To this end, brain-wide imaging with single-cell resolution is desired to simultaneously access morphological features of a neuron as well as to delineate the connectivity patterns of the neuron networks.

With the remarkable advances in sparse labeling [9–11], tissue clearing [12–14], light microscopes [15–18] and computational methods [19, 20], brain-wide mapping with single-cell resolution has become possible for small mammals, such as rodents. This has brought invaluable opportunities to understand the brain structures and the underlying mechanisms of brain diseases. Nonetheless, studies at larger scale and more detailed level are needed to explore a large variety of neuron types as well as to get more comprehensive understanding of neuron connectivity and projection patterns. More importantly, non-human primates (NHP) are getting more attentions as better experimental models of human cognitive functions and brain diseases considering the fundamentally different brain structures and behaviors between different species [21–23]. To investigate NHP and human brains in a similar way as rodents has become one of the most important tasks to date in neuroscience. Unfortunately, the approaches for rodents are not directly applicable to NHPs due to the much larger brain sizes, stringent limitations on the numbers, and the substantially increased individual variability of the brains [24]. Challenges remain from all aspects, i.e., sample preparation, imaging, and massive data processing. Tissue clearing in combination with light-sheet microscopy is particularly applicable for large-volume imaging, yet with compromised spatial resolution [14, 25, 26]. Clearing of human brain tissues is also notoriously challenging as the penetration depth of chemicals is strongly limited by the dense and opaque molecules accumulated over decades-age [27]. The block-face imaging methods, e.g., the serial two-photon tomography (STPT) [15, 28] and micro-optical sectioning tomography [16, 29, 30], do not rely on tissue clearing yet normally take several days to image an entire mouse brain with cellular resolution, which make them hardly scalable for much larger NHP or human brains. In addition, the processing and analyzing of petabytes (PB) of volumetric image data from primate brains is another critical challenge. High-throughput systems are in urgent need, not only for imaging itself, but also for sample preparation and data analysis that facilitate the brain-wide neuroscience at single-cell resolution.

**Fig. 1:**
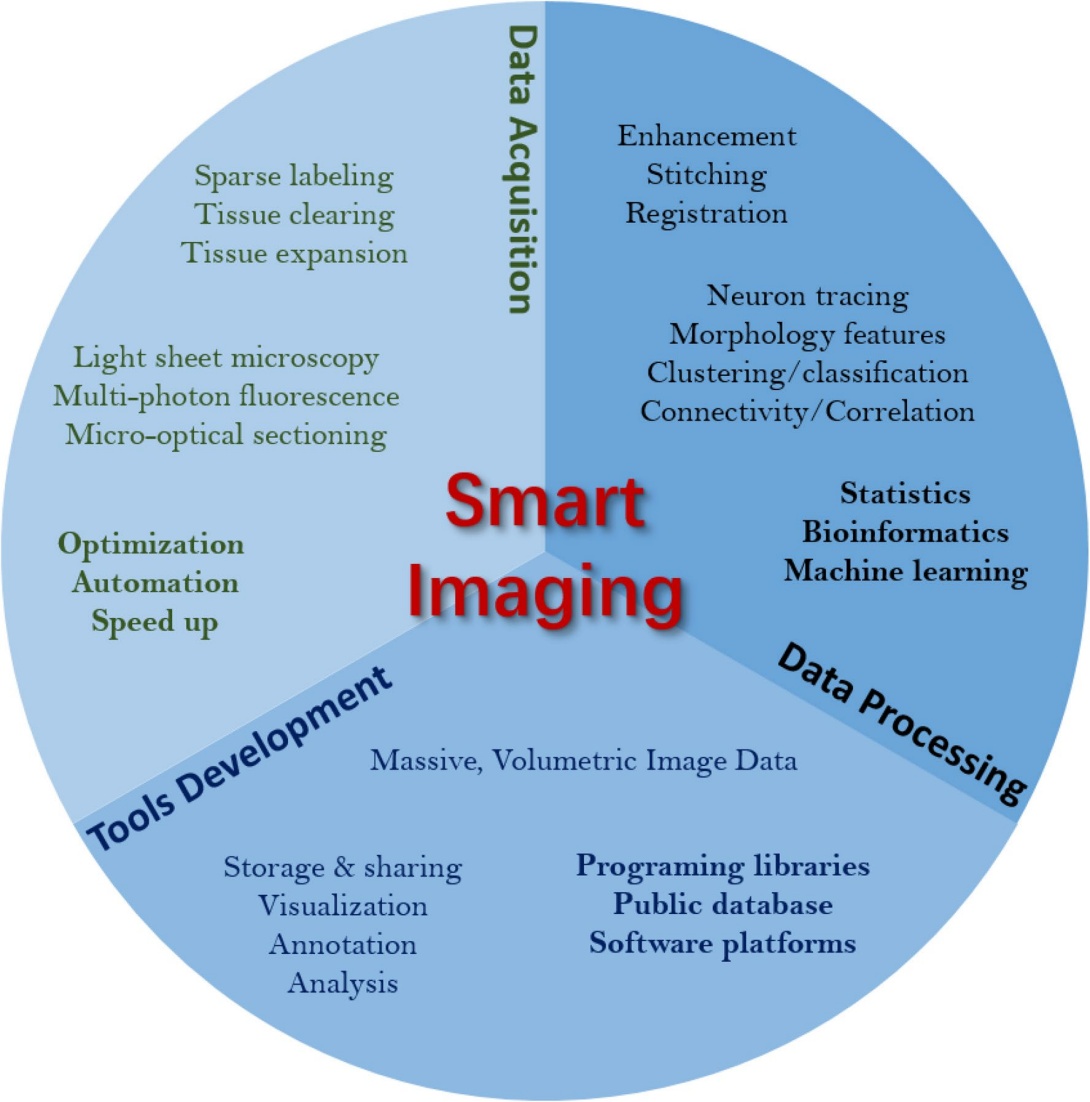
Key components to build up a smart imaging system in brain-wide neuroscience at single-cell level

Smart imaging systems emerge in this context (Fig. 1), in which the data acquisition and analysis are intensively supported by the artificial intelligence, software platforms, and computational facilities. All the three aspects are indispensable in a smart imaging system and strongly rely on each other. The data acquisition can be improved via automation to minimize error-prone manual operations and human interruptions. The information extracted from the images can be used to guide the data acquisition to reach better image quality at lower cost. The qualified data simplifies and very likely speeds up the subsequent data analysis and guarantees valid and meaningful biological information to be extracted. In addition, tools and platforms that support massive data management and analysis lower the barrier of carrying out analysis and developing new methods. Moreover, sophisticated manners of data sharing boost the world-wide cooperation; large-scale data annotation produces critical resource for method development and validation in artificial intelligence; data visualization provides a human–machine interaction for annotation, quality control, proofreading, etc. All such tools and platforms together can substantially speed up the advances of smart systems.

With all the considerations mentioned above, we investigate here the topics and techniques, ranging from sample preparation to data mining, that are considered critical to build up the smart imaging systems. We will start from the topic of data acquisition, including the sample preparation and optical imaging. This is followed with the discussion on data processing techniques, ranging from image preprocessing to data mining. The tools, platforms and database relevant to the above tasks will be summarized afterward. We will briefly touch on the growing waves of deep learning and cloud computing in neuroscience before conclusion. Note that we do not specifically limit the discussion on the field of neuroscience in this review, rather will include as well the techniques being used in other biological studies, with a hope to provide a broader view of smart imaging for neuroscience.

## Sample preparation

Sample preparation is perhaps the first major challenge to the imaging of large brains. It covers a variety of topics including sparse labeling, tissue clearing, tissue expansion. Sparse labeling plays an essential role to make the morphology of individual neurons well visible under an optical microscope. A different variety labeling methods and tracers are available and applied in combination with tissue clearing and expansion in brain imaging [11, 31]. Among those the genetic engineering and virus transfection provide rich information for the neuron labeling on animal models [9, 11, 32–34]. Simultaneous multi-color labeling and tracing of neurons is enabled by the brainbow AAVs [35].

Nonetheless, the labeling approaches mentioned so far are not applicable to human-brain analysis due to the ethical limitations. Labeling neurons by injecting dye, plasmid, or other markers into neurons is considered a powerful method in this regard. Despite the limitations to access long-range projections, dye injection in combination with surgery biopsies enables the investigation of human neurons of various cell types and from different brain regions based on local morphologies. To do so, the markers need to be injected into the cell body and transported to the axon terminals, for example, by alternative injection method, pressure injection or iontophoretic injection [36]. This provides a good way to target neurons of interest with sparsity, flexibility and specificity. Yet the procedure is very tedious, time consuming, and requires well-trained personnel. ‘Smart’ systems have been reported to automate and speed up the procedure. For instance, the ‘SmartACT’ was able to automatically guide the pipette to target the cell [37]; the automation was achieved for steps of pipette calibration, the target cell body targeting and the control of pipette movement in ‘Autopatcher IG’ [38]; A robotic system was applied to fill and move the pipette, and to break in the neuron cells [39]. Lately, a deep-learning based system was built up to detect and guide the pipette to approach, attach and break-in the neuron cells in vitro [40].

Following the sparse labeling, tissue clearing aims to change the optical properties of tissues to increase the penetration depth of optical imaging [12–14]. This can be achieved typically by replacing the lipid and water content of the tissue with a medium that has a refractive index matching the cellular content [12, 13]. In combination with light sheet microscopy, tissue clearing is playing a growing role in brain-wide imaging. Tissue expansion adopts the swellable polyelectrolytes to separate closely locating biological components [13, 41–43]. It is compatible with many labeling and clearing approaches and allows for nanoscale imaging with conventional microscopes. The spatial resolution of optical imaging can be substantially improved in this way. While tissue clearing and tissue expansion both are demonstrated successful in neuroscience, their performance is a mixture effect of the properties of samples and the imaging setups. To design and optimize the protocols for a specific study is pretty difficult and no smart systems, to our best knowledge, are adopted so far for these procedures. Yet we kindly refer to literatures [42, 44] in which critical guidance is presented with the pitfalls and approaches to optimal tissue clearing and expansion.

## Optical microscopy

**Fig. 2:**
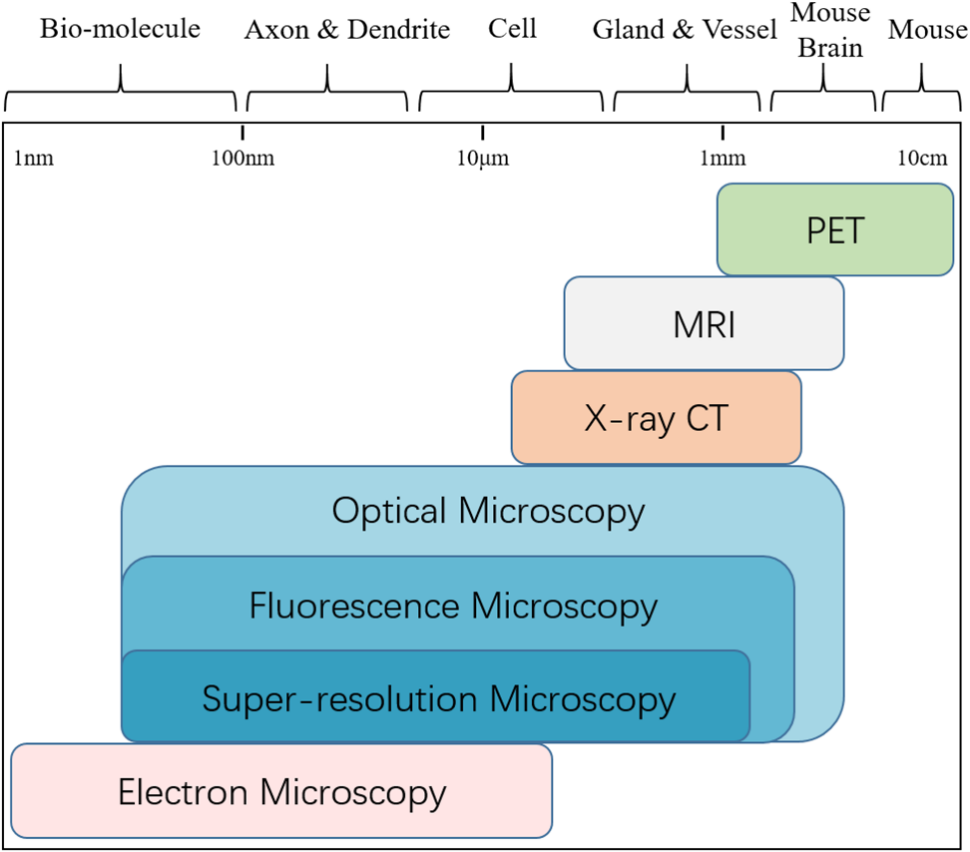
Different imaging modalities with respective to the spatial resolution

In combination with the labeling approaches, optical imaging makes up one of the most important toolsets in neuroscience, with unique advantages of spatial resolution to other imaging modalities (Fig. 2) [45]. Optical imaging has witnessed multiple technical innovations (see Table 1) [45–47] and is continuously empowered by structured light illumination [48], digital scanned LSFM [49], Lattice LSFM [50, 51], 2P-LSFM [52], etc. Many other techniques are reported as well, for instance, to speed up via the extended depth of field microscopy (EDOF) [53–55] or the customized chips [56–58], to improve the spatial resolution via the acousto-optic modulators or spatial light modulators [59, 60], to improve the axial accuracy via the depth sensors [61], to compensate the brain movements with the acousto-optic lens (AOL) 3D random-access pointing and scanning [62]. Aside from these hardware adaptions, AI techniques are vastly adopted to automate and speed up the imaging procedure as well as to improve the imaging quality [63–68]. For instance, to automatically adjust the illumination at real-time and minimize the need of imaging parameters tuning [64], to correct the aberrations using wave-front sensing method [65], to tackle the defocusing of the light sheet microscope with adaptive refocusing method [68]. In addition, content-aware imaging [63, 69, 70] and stitching [71, 72] are developed to suppress sample degradation, speed up the imaging, and enlarge the sample coverage. These advances have laid solid foundation for optical microscopy to be applied and adapted for brain-wide imaging with single-cell resolution, as is summarized in the following.

**Table 1:** Milestone innovations of imaging techniques

Techniques	Features	Achievements
CLSM [73]	Pin-hole structure to reject out-of-focus light	Improved axial resolution for optical sectioning
SDCM [74, 75]	Hundreds of pinholes arranged in spirals on an opaque disk that rotates at high speeds	Vastly speeds up image acquisition and reduce photon damage
2P microscopes [76]	Intense excitation by pulsed lasers, leading to absorption of two or three photons at once	Improved light collection efficiency, intrinsic confocal effect, and penetration depth
LSFM [77, 78]	Sheet-shaped excitation beam to selectively excite only the plane of interest	Faster imaging procedure, reduced photon damage
Multi-view LSFM [79, 80]	Simultaneously record multiple views of the specimen	Maximizes the sample coverage
LFM [81, 82]	A micro-lens array in place of the camera	Capture all voxels in a volumetric image instantaneously

*CLSM* confocal laser scanning microscope, *SDCM* spinning disk confocal microscopy, *2P* two-photon, *LSFM* light-sheet fluorescence microscopy, *LFM* light-field microscopy

To be applied in neuroscience, many efforts were made for a good balance between resolution, large volume and speed. In combination with sparse labeling and physical sectioning, the block-face systems enabled the 3D mapping of individual neurons across brain areas [83]. In particular, the micro-optical sectioning tomography (MOST) achieves sub-micro imaging of an entire mouse brain [29]. Serial two-photon tomography (STPT) achieves high-throughput fluorescence imaging of entire-brain in combination with an optical section in 50-micron-thickness tissue layers [28]. The fluorescence micro-optical sectioning tomography (fMOST) achieves micron imaging of entire mouse brain after fluorescent labeling and enables continuous tracing of neuronal circuits [84]. In combination with two-photon fluorescence and an acoustical optical deflector (AOD), moreover, the high-throughput two-photon MOST (2p-MOST) system obtained entire-brain imaging of ~ 0.32 µm × 0.32 µm × 1 µm resolution within 1 week [30]. The brain-wide positioning system (BPS) adopted multi-channel wide-field large-volume tomography (WVT) and acquired both labelled neural structures and cytoarchitecture reference in the same brain simultaneously [16]. The BPS system allows for precise localization of individual neurons and it takes 3 days for the entire brain imaging with ~ 0.32 µm × 0.32 µm × 2 µm resolution. Lately, a significant improvement in the penetration depth and background suppression was achieved in the HD-fMOST system via a line-illumination modulation (LiMo) technique [85]. In addition to the block-face systems, light sheet illumination shows unique advantage of high imaging throughput. Based on the light sheet microscopy, the entire-brain imaging at cellular resolution is achieved within a few hours for mouse brains [86, 87] and 100 h for monkey brains [17]. In combination with online image analysis, the sparsity property of neuron structures is highly implemented to speed up the imaging procedure [70, 71, 88]. As a new trend, the miniaturized imaging setups are being developed to bring new chances for in vivo brain science [89].

## Image preprocessing

Optical imaging is a mixture result of the optical properties of the sample and the setup. The influence of illumination, detector, lenses, etc. can introduce unavoidable yet significant contaminants into the raw images. Removing such destructing sources is one of the major tasks for image preprocessing. To this end, many image enhancement methods were developed, varying from filtering and rescaling to image transform, to achieve the aims of denoising [90–92], uneven illumination correction [93–96], deconvolution [97–99], etc. Many of the approaches, however, are not directly applicable to brain and neuron images, which contain rich tubular structures. In this regard, different approaches were proposed using the features of tubular structures characterized either by the eigenvalues of the local gradients or by the response of multi-directional filters [100–103]. The content-aware neuron image enhancement (CaNE) method [104], in particular, employed the properties of tubular structure in combination with the gradient sparsity of the neuron images. In addition, based on the sparsity of the neurites signals, the image enhancement was achieved by removing the background signal resulting from auto-fluorophores and substantially improved the subsequent neuron tracing [105].

Stitching is another task of image preprocessing encountered in brain imaging. Considering the large volume of brains and typically long-range projection of a neuron, imaging of multiple tiles and mostly also multiple tissue stacks is needed to get complete features of interest. This produces terabyte- and even petabyte-sized data sets comprised of many unaligned volumetric image tiles. Stitching is desired to reconstruct a complete volumetric image for further analysis. Therein, the globally optimal placement of image tiles is determined according to pre-defined quantities, such as the normalized cross-correlation and mutual information. For brain-wide imaging, approaches have been proposed particularly to deal with the massive data volume, including TeraStitcher [106] and BigStitcher [107] for overlapping tiles, and the custom software for non-overlapping tiles [17, 108]. Specifically, instead of stitching the image tiles, the NeuroStitcher proposed a way to assemble the neuron fragments after the neuron tracing from individual image tiles [109].

**Fig. 3:**
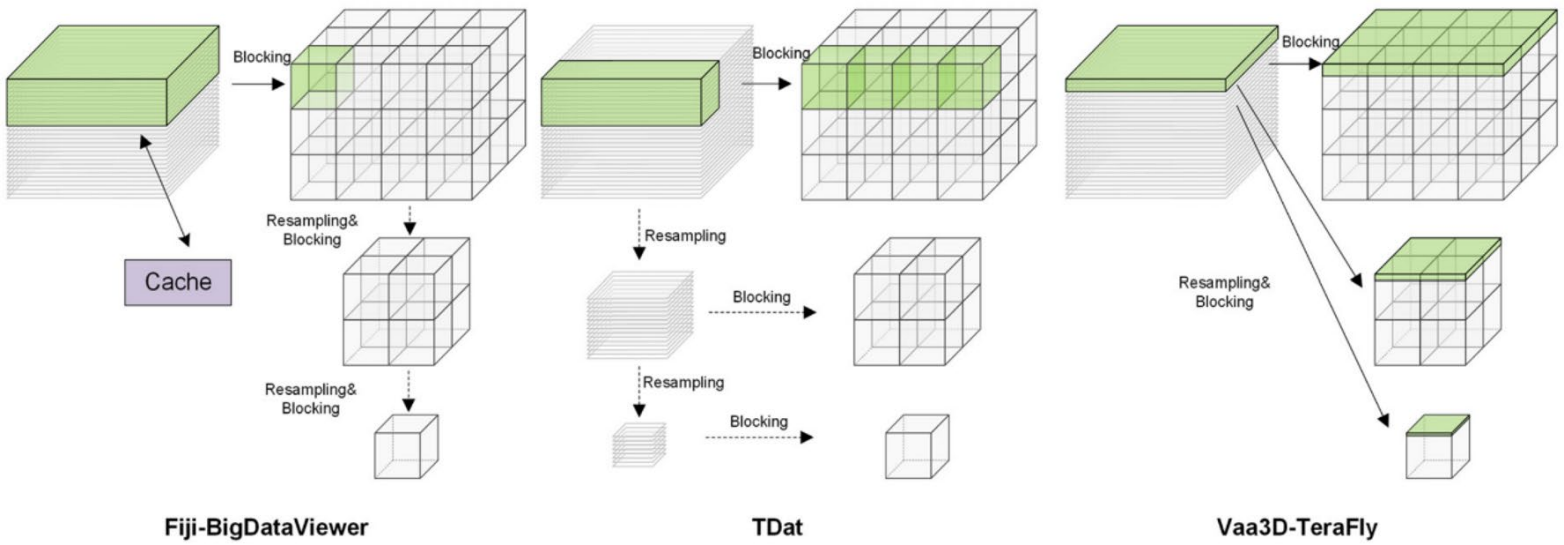
Principles of different data reformatting. BigDataViewer: the green blocks in the original space represent the data to be loaded into memory. One slice is read into memory once and cached. TDat: after recursively down-sampling the original data, only a CUBOID is read into memory and split into 3D blocks. Vaa3D-TeraFly: the data is read once and transformed in to multi-resolution (adapted from Ref. [110])

Another issue for image preprocessing comes to the re-slicing/reformatting to support the processing of large data volume. Herein the complete data volume is reformatted as blocks and mostly with hierarchical resolutions. The three typical data structures developed for this aim are depicted in Fig. 3 [110]. Therein the TeraFly [111] combined the pyramid image organization with the ‘mean and shift’ strategy to create smooth 3D exploration similar to ‘Google-earth’. The BigDataViewer [112] adopted caching mechanism for faster image reading. The TDat [110] read only cuboid data to control the memory consumption and sped up the data reformatting via distributed computation.

Last but not the least, registration is desired to align the entire-brain images from their respective coordinates to a standard brain space. This enables cross-brain and cross-modality analysis, as well as the analysis relative to brain regions and projection patterns. A common coordinate framework (CCF) for the mouse brain, in this regard, was built by co-aligning 1675 individual whole-brain data sets from STP tomography [113]. There 43 cortical areas, 330 subcortical gray matter areas, 82 fiber tracts, and 8 ventricle and associated structure volumes were all delineated natively in 3D. Registration methods were vastly investigated to map different brain data onto the reference atlas, including aMAP [114], ClearMap [115], qBrain [116], WholeBrain [117], SyN [118], etc. The procedure typically involves features/landmarks detection and image transformation [119]. As simple as it sounds, however, challenges exist, especially for the landmark detection, considering the variations in brain anatomy and intensity diversity caused by different sample preparation and imaging procedures. For this reason, a coherent landmark mapping (CLM) method was adopted to coherently deform the landmark points in the target image to find their best matches in the reference image [120]. The robustness of the registration is enhanced taking into consideration the brain regions segmented by a deep neural network. Nonetheless, the registration still requires semiautomatic refinement. Automatic registration, especially for TB- and PB-scale data is still an issue to conquer.

## Data mining

The aim of data mining is to extract information of interest from the image data and to finally draw biologically meaningful conclusions. In the field of neuroscience, and for the investigations of neuron circuits and connectivity in particular, major tasks of data mining include the neuron tracing and morphology analysis.

The shape of a neuron both determines and is constrained by its function and connection with other neurons. To understand and analyze the neuron morphologies hence plays a fundamental role in neuroscience. Neuron tracing is a critical step in this perspective [121–123]. It aims to create a digital reconstruction of the soma, dendrites, axon, and other sub-cellular components (e.g., spines, boutons, etc. [124, 125]) of a neuron. The traced neuron morphology are typically represented as a connected tree with the soma as the root and saved as SWC files, where each row gives the type, coordinates, radius/diameter, and parent of a node. Promoted by the DIADEM challenge [126], considerable efforts were made over the past years for automate neuron tracing with the aims to improve the speed, accuracy and reproducibility. Existent algorithms are normally composed of the elemental procedures including skeletonization [127, 128], seed generation [129], graph algorithms [130], deformable curves [131], image transforms, such as gradient vector field [127, 130], or learning-based approaches with annotated training data [129, 132]. Specifically, the tracing can be either obtained according to the information of the whole image [127, 133], or by exploring the image within the neighborhood of relevant structures [134, 135], categorized as global and local methods, respectively. On top of the basic algorithms, mechanisms are also introduced for the large-scale neuron tracing, such as the TReMap [136], UltraTracer [137], G-Tree [138], etc. A comprehensive summary of existent algorithms is available from ref. [123]. With the goal to “define and advance the state of the art of single neuron reconstruction, develop a tool-kit of standardized reconstruction protocols, analyze neuron morphologies, and establish a data resource for neuroscience”, the BigNeuron project [20] was jointly launched by several well-known brain research institutions. Therein numerous algorithms [130–132, 136, 139–142] were collected and evaluated with the ultimate goal to reach standard and unambiguous neuron tracings. Nonetheless, the automatic neuron tracing is still far from sophistication. It is hardly possible to trace single neurons without any human intervention at current stage. Manual inspection and correctness are always necessary post automatic tracing to remove errors, such as missing arbors, loops, trifurcations, etc. Last but not the least, the correctness of a neuron reconstruction is hard to justify considering the varying signal-to-noise ratio or the inadequate spatial resolution of the imaging.

Following the neuron tracing, to comprehensively analyze the traced morphometry data is critical to unravel the spatial properties of neurons and networks at multiple scales and to understand the mechanisms behind the nervous systems [143–145]. Many techniques have been developed for this aim [146, 147]. Among those the morphological grouping has been vastly applied, with the support of many similarity analysis [148–150] and clustering methods, such as UMAP [151], K-Means [152], and HCA [153]. In particular, morphological features such as L-measure were defined to quantify the morphological properties of neurons [154]. This in combination with machine learning and statistical analysis has been applied in cell typing, cross-brain-areal correlation analysis, etc. [3, 155–157]. With the help of a standard brain space, moreover, neurons were also classified according to the projection patterns [158]. Furthermore, a sequence representation was proposed to characterize the topologies of a neuron and successfully used for neuron classification [159, 160]. As a growing trend, the morphological analysis is being combined with other data modalities such as genomics to achieve better understanding in brain functions, such as cross-species comparison [161]. Nonetheless, the morphological analysis is still in its infancy. How to characterize the morphology remains to be a key bottleneck. There is a long way to adopt more techniques from statistics and machine learning.

## Tools, platforms, and database

The advances of smart systems largely benefited from the open-source tools and platforms, which have enabled researchers to reuse existing techniques and easily scale up or develop custom analysis strategies. This is not limited to the many programing libraries such as VTK, ITK, and OpenCV for general image analysis and Caffe, Keras, Tensorflow, PyTorch, and Theano for deep learning [162, 163]. Integrated platforms are also being developed [164–169] to lower the barrier of data analysis. Their featured functions and URLs are listed in Table 2. Along with the commercial software including Amira [170], Imaris (Bitplane Scientific Software), Neurolucida [171], and Image Pro (MediaCybernetics), these platforms have enabled the biologists and neuroscientists to conduct data analysis in an easier and more friendly way. In particular, the trees toolbox [172] provides a great platform for morphological analysis of individual neurons in isolation. The Natverse [173] was reported as a suite of R-packages for large-scale neuronal data processing, including the functions from local/remote data import, visualization, data transformation across template spaces, clustering and graph-theoretic analysis of neuronal branching. Vaa3D as a platform of big biological data analysis and computation has involved the many functions of data annotation, visualization, registration, neuron reconstruction and morphological analysis, etc. [111, 174, 175]. Tools for massive data storage, visualization, annotation, and indexing are being constructed worldwide as well. Additional to the hierarchical data structures of BigDataViewer [112], TeraFly [111] and TDat [110], for example, the Open Microscopy Environment’s Remote Objects (OMERO) and the Bio-Image Semantic Query User Environment (BISQUE) [176, 177] are constructed to facilitate the data annotation and content based data searching. Tools such as VirtualFinger [178], TeraVR [175], etc. boost the manual neuron tracing, data annotation and proofreading by creating an intuitive and immersive environment. Additional to these software platforms, public data repositories are being released with collective efforts over the world. The recently released database Morphohub [179], for example, enables the petabyte-scale multi-modal morphometry data storage, sharing, and analysis [179]. Image databases in the field of brain and neuroscience are summarized in Table 3, covering the various species from mouse to human and non-human primates. With the growing awareness to share and document data, public database starts to play a critical role for the development and validation of a new method as well as the comparison between different analysis methods.

**Table 2:** Typical open-source platforms for data processing

Software	Featured functions	URL
ImageJ [180]	Multi-purposed tool for image visualization and analysis; rich user-developed plugins	https://imagej.nih.gov/ij/
BioImageXD [181]	2D and 3D analysis; immersive visualization of multidimensional data	http://www.bioimagexd.net/
Icy [182]	Visualize, annotate, and quantify 2D and 3D bioimaging data	http://icy.bioimageanalysis.org/
FarSight	Display results derived from segmentation, tracking, feature extraction; build connections between these results and the raw data	http://farsight-toolkit.org/
FluoRender [183]	Data visualization and analysis; support multi-channel volume data and polygon mesh data rendering	https://github.com/SCIInstitute/fluorender/releases
BisQue [177]	Server-based platform for image sharing, analysis, visualization, and organization	https://bioimage.ucsb.edu/bisque
Trees toolbox [172]	Edit, visualize and analyze neuronal trees; neuron reconstruction; generation of synthetic neuron morphologies	http://www.treestoolbox.org
GTree [138]	System for brain-wide neuron tracing and error-screening	https://github.com/GTreeSoftware/GTree
Lychnis [17]	3D neuron tracing, interactive visualization and annotation	https://doi.org/10.1038/S41587-021-00986-5
Vaa3D [174]	Registration; real-time visualization and analysis of large-scale multidimensional data; (brain-wide) neuron reconstruction; content extraction and annotation directly in 3D; rich user-developed plugins;	http://www.vaa3d.org/
Natverse [173]	Local/remote data import, visualization, data transformation/registration, clustering and graph-theoretic analysis of neuronal branching	https://natverse.org/
SNT [184]	Neuron tracing, proof-editing, and visualization; Morphological quantification and modeling	https://github.com/morphonets/SNT

**Table 3:** Open database in neuroscience, which contains multiple species as highlighted in bold

Database	Description	Data type
Allen Brain Atlas [185]	Atlas, stained sections from **mouse and human** brains indicating development and gene expression	Images
BrainMaps [186]	Atlas, high resolution stained sections from brains of **14 species including human and NHP**	Images
Brain Biodiversity Bank (brains.anatomy.msu.edu/museum/brain)	Atlas, stained sections and MRI images from brains of **human and 62 other species**	Images
Cerebellar Development Transcriptome Database [187]	Atlas, stained sections from **mouse** brains indicating cerebellar development and gene expression	Images
Whole Brain Atlas [188]	Atlas, structural MRI images and PET images of **human** brains	Images
Mouse Brain Library [189]	Atlas, stained sections from **mouse** brains	Images
Neuromorpho [190]	3D models of neurons from **human, rat, mouse, monkey, and others**	Images and 3D data

## Growing trends

**Fig. 4:**
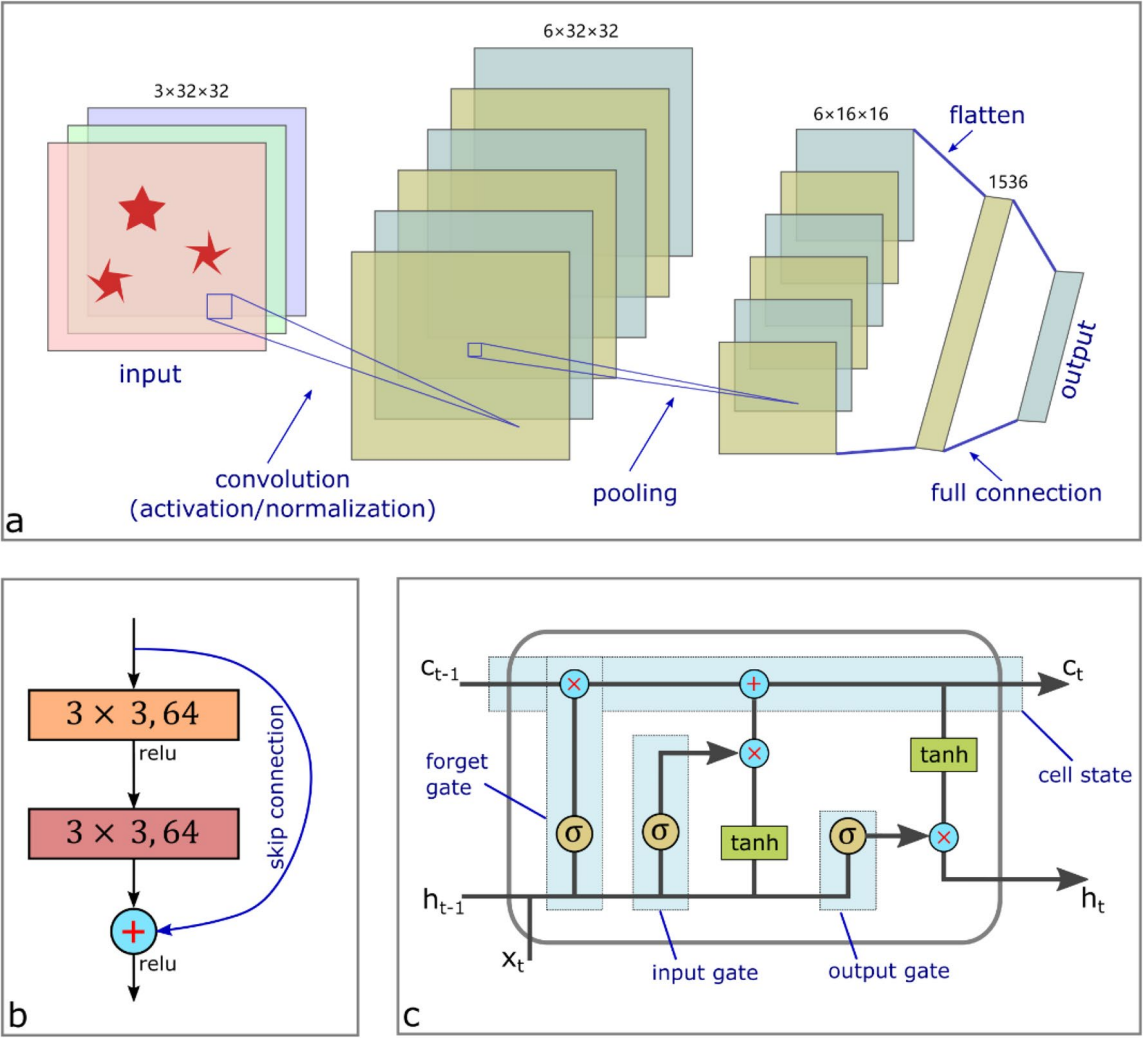
Building blocks of **a** CNNs, **b** ResNet, and **c** LSTM

The last two decades witnessed the prosperity of deep learning in biological data analysis and neuroscience, under the support of the variety of network architectures. Among those the convolutional neural network (CNN) and recurrent neural network (RNN) are the most commonly used. In general, CNNs are mostly employed for image analysis and computer vision, while RNNs are more applied in time-series problems. Many networks of these two kinds were developed in the past several years: the AlexNet [191], ResNet [192], Inception [193, 194], DenseNet [195], VGG [196], DCGAN [197], GoogleNet [198] for CNN and LSTM [199], Bi-RNN [200], and GRU [201] for RNN. Typically, CNN is composed of a series layers including convolution (followed by activation and normalization), pooling (for sub-sampling), and fully connected layers (Fig. 4a). The flatten and fully connected layers are removed in full convolution networks (FCN), which is particularly useful if the size of input data varies from time to time. ResNet as a typical CNN is made up of residual blocks (Fig. 4b), where a skip connection is adopted to deal with the gradient vanish problem and hence enables to train deeper networks. The LSTM as a typical example of RNN consist of the blocks of memory cell state (Fig. 4c) that are regulated by the input, forget and output gates. This helps to retain knowledge of earlier states and partly addresses the problem of vanishing gradients.

Deep learning brings vast opportunities. Image preprocessing is more and more achieved via deep learning [202]. For example, the CARE [203] for super-resolution wide-field images [204, 205] and the VCD on a light-field microscopy for artifact-free volumetric imaging with uniform spatial resolution at video-rate [206]. Unsupervised networks such as N2V [207], PN2V [208], Noise2noise [209, 210], Noise2Self [211] and Cyclegan [212] have shown to compete the supervised networks in denoising tasks. Promising results of deep learning are demonstrated for registration [120, 213, 214]. 3D segmentation is well achieved via 3D neural networks, such as CDEEP3M [215] and Cellpose [216]. Rapid 3D neuron tracing was achieved via the circulating neural network based on flood algorithm for the 3D image segmentation [217]. The networks of DeepNeuron [218] and SmartTracing [132] were proven promising in neuron reconstruction. The advances in natural language processing and the networks such as transformer [219, 220] along with the sequence representation [159, 160] of neuron morphologies has provided promising approach to cell typing particularly for full-neuron-morphology classification.

Cloud computing is another product of the fast advancing computational resources and internet [215, 221]. It allows a user to access and share data, applications, and infrastructures from a remote location. The most popular cloud services are Software as a Service (SaaS), Platform as a Service (PaaS), and Infrastructure/Hardware as a Service (IaaS/HaaS), with varying need of user management (see Table 4) [222]. With SaaS, the users are able to access software via a browser from the third-party provider without complex installation or hardware management. The PaaS provides platforms on the server so that a user can develop web applications without installing any tools. The IaaS means the provider shares the IT Infrastructure to users, which releases the need to purchase and maintain the infrastructure.

**Table 4:** Need of user management in different models of cloud computing

	SaaS	PaaS	IaaS
Applications	No	Yes	Yes
Data	No	Yes	Yes
Runtime	No	No	Yes
Middleware	No	No	Yes
O/S	No	No	Yes
Virtualization	No	No	No
Servers	No	No	No
Storage	No	No	No
Networking	No	No	No

The cloud-computing has made life much easier for data sharing, annotation and analysis, with smoother multi-user interaction and cooperation in remote and worldwide [222]. Moreover, server-based platforms have lowered the barriers of model construction, distribution and re-training. For example, cloud-based deep neural networks are being developed to release the users from the tedious configurations of deep learning environment [215]. The interactive machine learning platform ‘ilastik’ efficiently combines the annotation and model training; it thus allows to begin the model training with a small amount of annotated data and add more annotations interactively over the training steps [223]. A server-less web application ‘imJoy’ works across different systems and on both desktop and mobile devices [224]. It provides an easy-to-use data analysis tool that allows visualization, classification, deep learning, etc. All functions are provided as independent plugins that can be built using different programming languages. The ‘NeuroCAAS’ is constructed as a cloud-based platform for the data analysis in neuroscience [221]. Through the drag-and-drop interface, users can simply choose and configure the algorithms available from the platform. The requested analysis is then automatically deployed as a ‘blueprint’ and performed by the platform. Taking advantages of all these techniques, a ‘laboratory as a server’ could not be far away, in which researchers can control and share the imaging equipment from remote. All such cloud-based platforms together will bring vast opportunities to facilitate artificial intelligence and smart systems, and further promote our exploration of brain-wide neuroscience at single cell level.

Last but not the least, the technologies discussed so far are being increasingly combined into integrated systems to play a larger role than they can do individually. Therein, the computational technologies, resources and internet are considered the muscles and blood vessels on top of the hardware skeletons to smoothly combine data acquisition, management and analysis [17]. With minimal human interruption, such integrated systems can intrinsically minimize human errors and improve the throughput. In addition, the closed loop from data acquisition to analysis helps largely to improve the performance of a system. More importantly, an integrated system shows collective wisdom from experts of multiple disciplines (e.g., neuroscientists, physicists, computer scientists, etc.) and worldwide to ensure ‘optimal and correct’ output of the system. With carefully designed pipelines, therefore, integrated systems are to become another trend in the current era of big science and play an essential role in brain research. With modules such as optical sectioning, image acquisition, data reconstruction and analysis, etc. all combined together, the past years have witnessed several integrated systems being developed for brain research [16, 17, 87, 88]. This has led to many exciting discoveries and particularly leveraged the studies on NHP and human brains [1, 2, 17]. With continuously advancing AI technologies, we expect more smart integrated systems being established to push brain research towards a new milestone.

## Conclusions

To conduct brain-wide imaging at single-cell resolution for non-human primates and humans has become an important task in neuroscience. This is expected to produce similar discoveries as it was for rodents and finally lead to deeper understanding of the structures and connectivity of human brains. High-throughput imaging systems are in urgent demand considering the large brain sizes. Smart systems empowered by AI techniques and computational resources show huge potential to this end. In this review, we investigated the AI techniques that have been or can be applied in neuroscience, ranging from the tasks of sample preparation, image acquisition and analysis. We also discussed the software tools and database that can facilitate the development of AI techniques and smart systems. By absorbing more AI techniques and taking advantages of the super computational resources, such as deep learning and cloud computing, apparently, the smart systems supporting ‘super’ high-throughput imaging and scalable massive data processing will certainly play an invaluable role for neuroscience to reach deeper and broader knowledge on brain structures and connectivity.

## Data Availability

Data sharing is not applicable to this review article as no new data were created or analyzed in this study.
